# Two novel *Enterobacter* species, *Enterobacter chinensis* sp. nov. and *Enterobacter rongchengensis* sp. nov., recovered from clinical samples carrying multiple virulence factors

**DOI:** 10.1128/spectrum.00292-24

**Published:** 2024-06-25

**Authors:** Yanling He, Yuling Xiao, Yu Feng, Shikai Wu, Li Wei, Zhiyong Zong

**Affiliations:** 1 Center of Infectious Diseases, West China Hospital, Sichuan University, Chengdu, China; 2 Division of Infectious Diseases, State Key Laboratory of Biotherapy, Chengdu, China; 3 Center for Pathogen Research, West China Hospital, Sichuan University, Chengdu, China; 4 Laboratory of Clinical Microbiology, Department of Laboratory Medicine, West China Hospital, Sichuan University, Chengdu, China; 5 Department of Infection Control, West China Hospital, Sichuan University, Chengdu, China; University of Torino, Turin, Turin, Italy

**Keywords:** *Enterobacter*, *Enterobacteriaceae*, Enterobacterales, taxonomy, *Enterobacter chinensis*, *Enterobacter rongchengensis*

## Abstract

Two *Enterobacter* strains 170198^T^ and 170250^T^ were isolated from clinical blood samples from distinct patients in a hospital in Chengdu, China, in 2022. These isolates were subjected to whole-genome sequencing. A phylogenomic tree based on 2,096 concatenated core genes showed that the two strains were clustered within the genus *Enterobacter*. The average nucleotide identity (ANI) and *in silico* DNA–DNA hybridization (*is*DDH) values between each of the two strains and type strains of all currently known *Enterobacter* species were determined. The two strains belonged to two novel species as the highest ANI and *is*DDH values with type strains of all currently known *Enterobacter* species below the cutoff for species demarcation (96% for ANI and 70% for *is*DDH). Then the physiological and biochemical studies demonstrated that biochemical features and the profile of whole fatty acids of strains 170198^T^ and 170250^T^ were largely consistent with those known *Enterobacter* species. Nevertheless, the two novel species can be differentiated from all other *Enterobacter* species by certain biochemical characteristics. In conclusion, 170198^T^ and 170250^T^ represent two novel species of the genus *Enterobacter*, for which we propose *Enterobacter chinensis* sp. nov. and *Enterobacter rongchengensis* sp. nov., as the species names. The type strains of *Enterobacter chinensis* sp. nov., and *Enterobacter rongchengensis* sp. nov. are 170198^T^ (=GDMCC 1.3549^T^=JCM 35826^T^) and 170250^T^ (=GDMCC 1.3670^T^=JCM 36189^T^), respectively. The two novel species have clinical significance with the ability to cause bloodstream infections.

**IMPORTANCE:**

*Enterobacter* is a group of bacteria comprising several common opportunistic pathogens and has a complicated taxonomy. Here, we reported two novel *Enterobacter* species. We demonstrated that the two novel species can be differentiated from other *Enterobacter* species by certain phenotypic characteristics and therefore provide information for designing tests for identification. We also showed that strains of the two novel species are able to cause human bloodstream infections and carry multiple virulence factors and therefore are of clinical significance. We highlight that the virulence of *Enterobacter* is less studied and warrants further exploration. We believe that the findings here are valuable for enhancing the appreciation toward *Enterobacter*, an important pathogen.

## INTRODUCTION

*Enterobacter* is a genus of the family *Enterobacteriaceae*, which was first proposed by Hormaeche and Edwards (1). Members of the family *Enterobacteriaceae* have been reclassified in 2016, and currently, *Enterobacteriaceae*, together with families *Budviciaceae*, *Erwiniaceae*, *Hafniaceae*, *Morganellaceae*, *Pectobacteriaceae*, and *Yersiniaceae,* is under the order Enterobacterales (2). *Enterobacter* is widely distributed in nature, and some species are common nosocomial opportunistic pathogens causing a wide range of infections (3). Notably, *Enterobacter cloacae* complex is a common term used clinically and comprises several species sharing ≥60% overall nucleotide similarity of genomes (4–6). Currently, there are 23 species of the genus *Enterobacter* (7–9), and *Enterobacter* is closely related to the genera of *Huaxiibacter*, *Leclercia*, *Lelliottia*, and *Pseudoenterobacter* (10–12). Here, we reported two novel *Enterobacter* species via characterization of two stains causing bloodstream infection in hospitalized patients at our hospital in 2022.

## MATERIALS AND METHODS

### Strains and *in vitro* susceptibility

Strains 170198^T^ and 170250^T^ were recovered from the blood samples of two hospitalized patients as part of standard care. The strains were preliminarily identified by Vitek II automated system (bioMérieux, Durham, DC, USA) and using matrix-assisted laser desorption/ionization time-of-flight mass spectrometry (MALDI-TOF; Bruker, Billerica, MA, USA; MBI compass version 4.1.100). Antimicrobial susceptibility testing was performed using the broth microdilution method except for fosfomycin, which was determined using the agar dilution method, according to guidelines of the Clinical and Laboratory Standards Institute (CLSI) (13). Antimicrobial agents tested were amikacin, ampicillin, ampicillin–sulbactam, amoxicillin–clavulanate, aztreonam, cefazolin, cefepime, cefotaxime, ceftazidime, ceftriaxone, cefuroxime, cephalothin, ciprofloxacin, colistin, doripenem, ertapenem, fosfomycin, gentamicin, imipenem, levofloxacin, meropenem, moxifloxacin, piperacillin, piperacillin–tazobactam, tetracycline, ticarcillin, tigecycline, tobramycin, and trimethoprim/sulfamethoxazole. The susceptibility results were also interpreted based on CLSI except those of moxifloxacin, ticarcillin, and tigecycline, which were based on European Committee on Antimicrobial Susceptibility Testing (http://www.eucast.org/), considering the absence of CLSI breakpoints and categories for these agents.

### 16S rRNA gene sequence analysis

The nearly complete 16S rRNA gene sequence (1,465 bp) of the strains were obtained using PCR with primers 27F and 1492R (14) and subsequent Sanger sequencing. The 16S rRNA gene sequences were compared with those of type strains of all known species belonging to the genera of *Enterobacter*, *Huaxiibacter*, *Leclercia*, *Lelliottia*, and *Pseudoenterobacter* using BLAST (https://blast.ncbi.nlm.nih.gov/Blast.cgi).

### Whole-genome sequencing and phylogenomic analysis

Genomic DNA of strains 170198^T^ and 170250^T^ was prepared using the QIAamp DNA mini kit (Qiagen, Hilden, Germany) and subjected to genome sequencing using the HiSeq X10 Sequencer (Illumina, San Diego, CA, USA) according to the manufacturer’s instructions. Paired-end 150-bp reads were trimmed for removing adapters using Trimmomatic v0.39 (15) and were then assembled to contigs using SPAdes v3.15.3 (16). Type or reference strains of species within the family *Enterobacteriaceae* were retrieved from GenBank (https://www.ncbi.nlm.nih.gov/genbank/). For the raw sequence data, quality control, trimming, and genome assembly were performed using Shovill v1.1.0 (https://github.com/tseemann/shovill). All the genomes were annotated using Prokka v1.14.5 (17) with default settings. A pan-genome consisting of core genes with a ≥50% coverage of encoded amino acids in all the above genomes was constructed using PIRATE v1.0.4 (18). Core-genome single-nucleotide polymorphisms (SNPs) were extracted from the alignment file generated by PIRATE using SNP-sites v2.5.1 (19). The phylogenomic tree was then inferred using IQ-tree v2.1.2 (20) under the GTR model with γ rate heterogeneity (+G), ascertainment bias correction (+ASC), and 1,000 ultra-fast bootstraps. We also inferred a phylogenomic tree based on The Genome Taxonomy Database (GTDB, https://gtdb.ecogenomic.org/).

### Average nucleotide identity (ANI) and *in silico* DNA–DNA hybridization (*is*DDH) analysis

Pairwise average nucleotide identity (ANI) and *in silico* DNA–DNA hybridization (*is*DDH) values were determined between each of the strains and the type or reference strains of all known species within the genus *Enterobacter* and the closely related genera *Huaxiibacter*, *Leclercia*, *Lelliottia*, and *Pseudoenterobacter* for species demarcation. ANI and *is*DDH values were calculated using FastANI v1.33 (21) and Genome-to-Genome Distance Calculator (formula 2) (22), respectively. Values of ≥96% ANI (23, 24) and ≥70% *is*DDH (25) were used as the cutoff to define species.

### Identification of antimicrobial resistance and virulence genes

Antimicrobial resistance determinants were predicted with ABRicate v1.0.0 (https://github.com/tseemann/abricate) using ResFinder databases. Virulence factors were identified using VFanalyzer to query the virulence factor database (http://www.mgc.ac.cn/VFs/) (26). The chromosome or plasmid location of genes was predicted using SourceFinder (27).

### Morphological observation and biochemical assays

A set of morphological and phenotypic experiments were performed to characterize the two strains. The cell morphology and motility of the strains were observed using a CX21FS1 light microscope (Olympus, Tokyo, Japan) and HT7800 transmission electron microscopy (Hitachi, Tokyo, Japan). Gram staining was performed as described previously (28). Bacterial growth was determined at different temperatures (4°C, 15°C, 20°C, 25°C, 30°C, 37°C, 40°C, 42°C, 45°C, and 50°C), at different pH values (3.0–12.0, at intervals of 1.0 pH), and at multiple concentrations of NaCl (0%–10%, wt/vol, at intervals of 1%) after a 2-day incubation in 15-mL test tubes containing 3 mL of tryptic soy broth (TSB; Hopebio, Qingdao, China) placed in a thermostatically controlled water bath as described previously (29). Anaerobic growth was examined using an anaerobic bag (bioMérieux) by incubating the isolates on nutrient agar (Sangon Biotech, Shanghai, China) for 7 days. Catalase activity was tested by dropping 3% (vol/vol) H_2_O_2_ on culture grown for 24 h on nutrient agar. Oxidase activity was determined using oxidase strips (Hopebio). Hemolysis was observed on blood agar plates. The physiological and biochemical properties were evaluated using the API 20E and API 50CH kits (bioMérieux) according to the manufacturer’s instructions. *Escherichia coli* strain ATCC 25922 was used as the control.

### Whole-cell fatty acid profiling

Whole-cell fatty acid profiles were determined by Guangdong Institute of Microbiology (Guangzhou, China) as described previously (30).

## RESULTS

Strains 170198^T^ and 170250^T^ were recovered from the blood samples of two hospitalized patients with bloodstream infections. Given that no other pathogens were detected from the blood of the two patients during the disease duration, the two strains were determined as the pathogen causing bloodstream infection. The two patients were in an immunocompromised status, with neutropenia due to cancer chemotherapy or receiving immunosuppressants after a renal transplantation. Both patients had fever but did not develop septic shock and recovered uneventfully after antimicrobial chemotherapy, meropenem in one patient and levofloxacin in another. The two clinical strains were identified as *E. cloacae* complex via conventional identification using the Vitek II and as *Enterobacter bugandensis* using MALDI-TOF. To determine the exact species of the isolates, we obtained the nearly complete sequence of the 16S rRNA gene of the two strains and found that the 16S rRNA sequence of 170198^T^ and 170250^T^ shared the maximum identity with *Enterobacter kobei* (99.92%) and *Huaxiibacter chinensis* (98.11%), respectively. Considering the low resolution of 16S rRNA gene analysis for species identification (31), we performed whole-genome sequencing for the two strains and phylogenomic analysis based on core genes.

**Fig 1:**
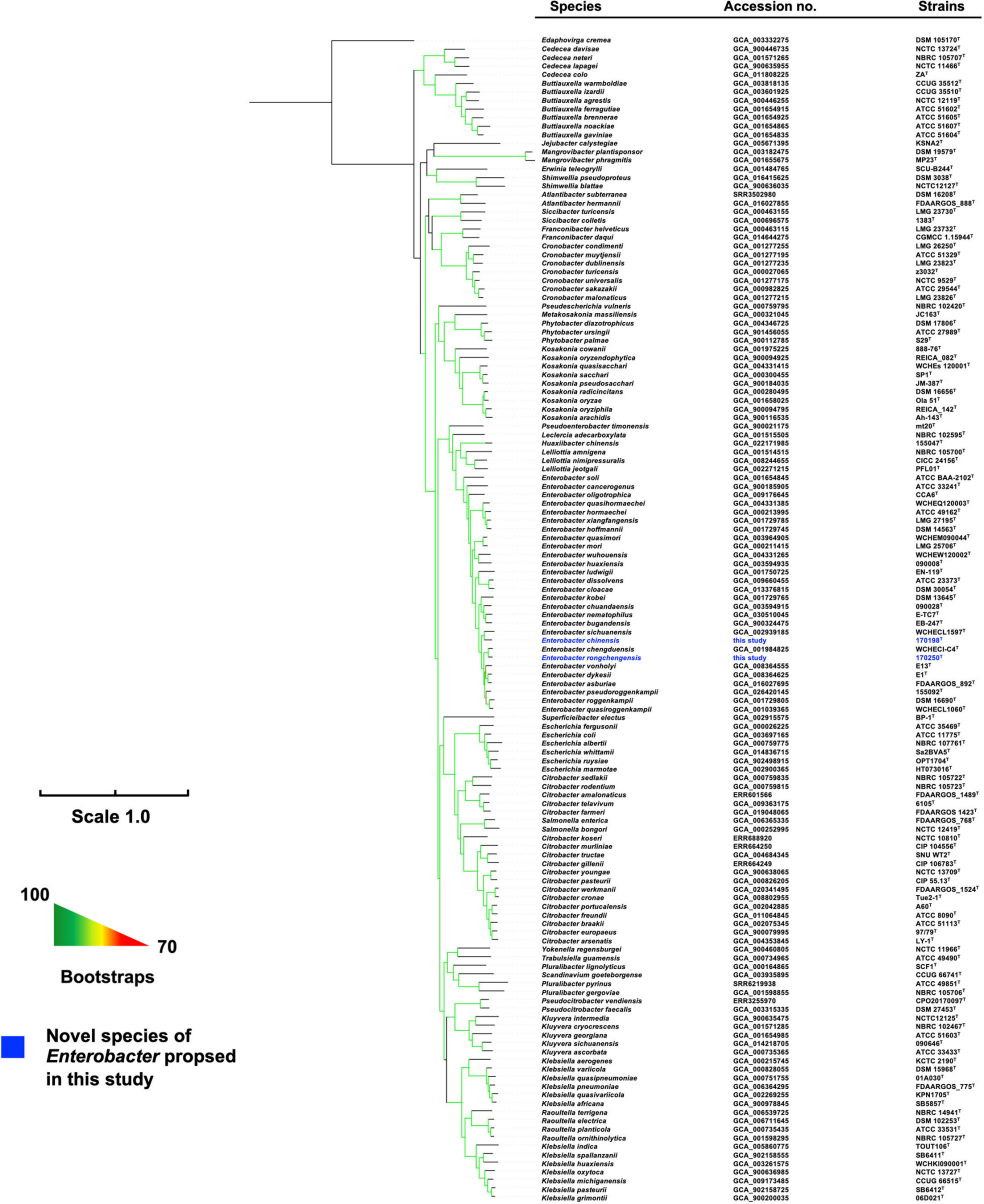
Maximum likelihood phylogenomic tree based on the concatenated nucleotide sequence of core genes of strain 170198^T^, 170250^T^, and type strains of the genera *Enterobacter*, *Huaxiibacter*, *Leclercia*, *Lelliottia*, and *Pseudenterobacter*. Bootstrap values (>70%) after 1,000 resamplings are shown by color gradients. Scale, 10% nucleotide sequence divergence.

Whole-genome sequencing generated a total of 10,047,104 [1.51 gigabases (Gb)] and 12,662,322 (1.90 Gb) reads for strain 170198^T^ and 170250^T^, respectively, with a 200× coverage. The draft genome of strain 170198^T^ was 4.9 Mb containing 28 contigs (≥200 bp; N*50*, 702,505 bp) with a 55.12 mol% DNA G + C content. The 4.9 Mb draft genome of strain 170250^T^ contains 89 contigs (≥200 bp; N*50*, 199,762 bp; 56.18 mol% G + C content). The GTDB tree indicated strains 170198^T^ and 170250^T^ were located within the *Enterobacter* cluster (Fig. S1 in the Supplementary material). However, this phylogenomic analysis only utilized 120 core genes that reflect species characteristics with a relatively lower resolution (32). We therefore inferred another phylogenomic tree based on 2,096 core genes, which also demonstrated that strains 170198^T^ and 170250^T^ were located within the genus *Enterobacter*, most closely to *Enterobacter sichuanensis* and *Enterobacter chengduensis*, respectively (Fig. 1). Strain 170250^T^ had the highest ANI (92.93%) and *is*DDH (49.9%) values with the type strain of *E. chengduensis* (accession no. GCA_001984825) (Table 1). Strain 170198^T^ had the highest ANI (95.58%) and *is*DDH (64.4%) values with the type strain of *Enterobacter asburiae* (accession no. GCA_016027695) (Table 1).

**TABLE 1:** Average nucleotide identity (ANI) and *in silico* DNA–DNA hybridization (*is*DDH) values between strains 170198^T^, 170250^T^, and type strains of the genus *Enterobacter* and the closely related genera *Huaxiibacter*, *Leclercia*, *Lelliottia*, and *Pseudoenterobacter^a^^,b^*

Strain	Accession no.	ANI/*is*DDH (%)	
		170198^T^	170250^T^
170198^T^	JARDVI000000000	-	91.98/45.6
170250^T^	JARDVK000000000	87.81/45.6	-
*E. asburiae*	GCA_016027695	**92.93/49.9**	93.3/52.1
*E. bugandensis*	GCA_900324475	90.78/41.0	91.13/42.1
*E. cancerogenus*	GCA_900185905	92.79/31.5	87.25/31.7
*E. chengduensis*	GCA_001984825	86.95/45.3	**95.58**/**64.4**
*E. chuandaensis*	GCA_003594915	91.74/41.5	91.19/42.2
*E. cloacae*	GCA_013376815	90.97/36.3	88.54/35.4
*E. dissolvens*	GCA_009660455	89.07/36.4	88.8/35.4
*E. dykesii*	GCA_008364625	89.25/48.3	93.17/50.6
*E. hoffmannii*	GCA_001729745	92.66/32.9	88.03/33.6
*E. hormaechei*	GCA_000213995	87.68/33.0	88.06/33.5
*E. huaxiensis*	GCA_003594935	87.72/34.3	88.65/34.4
*E. kobei*	GCA_001729765	88.48/40.5	90.81/41.8
*E. ludwigii*	GCA_001750725	90.49/35.2	88.37/34.7
*E. mori*	GCA_000211415	88.73/37.4	89.66/37.2
*E. nematophilus*	GCA_026344075	91.04/41.8	91.45/43.1
*E. oligotrophica*	GCA_009176645	89.87/33.6	87.94/33.4
*E. pseudoroggenkampii*	GCA_026420145	88.04/48.7	93.33/51.3
*E. quasihormaechei*	GCA_004331385	87.66/32.8	87.99/33.5
*E. quasimori*	GCA_003964905	90.02/37.7	90.32/38.7
*E. quasiroggenkampii*	GCA_001039365	92.39/47.4	93.08/50.2
*E. roggenkampii*	GCA_001729805	92.40/47.3	92.9/49.3
*E. sichuanensis*	GCA_002939185	92.51/47.7	91.03/42.6
*E. soli*	GCA_001654845	86.75/30.8	86.47/30.4
*E. vonholyi*	GCA_008364555	92.38/47.2	92.91/49.5
*E. wuhouensis*	GCA_004331265	88.80/35.2	89.04/35.7
*E. xiangfangensis*	GCA_001729785	87.81/33.1	88.11/33.6
*Huaxiibacter chinensis*	GCA_022171985	84.03/25.4	83.94/25.9
*Leclercia adecarboxylata*	GCA_001515505	84.07/25.8	84.23/26.2
*Leclercia pneumoniae*	GCA_017348915	83.95/25.8	84.22/26.3
*Lelliottia amnigena*	GCA_001514515	83.70/25.5	83.64/25.5
*Lelliottia jeotgali*	GCA_002271215	84.56/26.8	84.64/26.8
*Lelliottia nimipressuralis*	GCA_008244655	84.75/26.6	84.87/26.9
*Pseudoenterobacter timonensis*	GCA_900021175	83.72/25.6	84.12/26.1

^*a*^ The closest matches are highlighted in bold.

^*b*^ -, not applicable.

Biochemical characteristics of strains 170198^T^ and 170250^T^ are shown in Table S1 in the Supplementary material and were compared with those of type strains of species of the genera *Enterobacter*, *Huaxiibacter*, *Leclercia*, *Lelliottia*, and *Pseudoenterobacter* (3, 7, 8, 10, 12, 29, 33–39) (Table S2 in the Supplementary material). The two species can be distinguished from each other and all other *Enterobacter* species by certain characteristics, which are described in the following species description sections. The major cellular fatty acids of 170198^T^ and 170250^T^ are C_16:0_, C_17:0_cyclo, C_18:1_ω7c, and C_16:1_ω7c/_C16:1_ω6c, which is consistent with other *Enterobacter* species (Table S3 in the Supplementary material).

The two strains were susceptible to amikacin, aztreonam, cefepime, ceftriaxone, ciprofloxacin, doripenem, ertapenem, gentamicin, imipenem, levofloxacin, meropenem, moxifloxacin, piperacillin, piperacillin–tazobactam, tetracycline, tigecycline, and tobramycin but were resistant to amoxicillin–clavulanate, cefazolin, cephalothin, and trimethoprim/sulfamethoxazole (Table S4 in the Supplementary material). In addition, strain 170250^T^ was resistant to ampicillin, ampicillin–sulbactam, cefotaxime, cefuroxime, and ticarcillin, and was intermediate to ceftazidime and colistin (Table S4). In contrast, strain 170198^T^ was resistant to colistin and fosfomycin and was intermediate to ampicillin (Table S4).

Four antimicrobial resistant genes, AmpC β-lactamase-encoding *bla*_ACT_, fosfomycin-resistance gene *fosA*, and *oqxA-oqxB* associated with reduced susceptibility to quinolones, all of which are intrinsic, chromosomally located to *Enterobacter*, were identified from the genome of strains 170198^T^ and 170250^T^. Notably, 170250^T^ has *bla*_ACT-48_, while 170198^T^ has a variant of *bla*_ACT_ encoding a novel ACT of a highest amino acid identity (94.23%) with ACT-6. The differences of ACT β-lactamases in the two strains may provide an explanation for the discrepancy of their susceptibility to certain β-lactams. A variety of virulence factors were identified in the genome of both strains 170198^T^ and 170250^T^ including aerobactin siderophore encoding the gene *iucABCD-iutA*, enterobactin encoding the *ent* gene cluster, type 1 fimbriae encoding *fim* operon, and type three fimbriae encoding the gene *mrkABCDF*, hemorrhagic *E. coli* pilus (HCP) encoding the gene *hcp*, curli fiber encoding the gene *csg*, and components encoding the genes of type VI secretion system. In addition, strain 170198^T^ harbored genes for endotoxin synthesis and a capsular polysaccharide (CPS) locus (40), while strain 170250^T^ had genes encoding multiple toxins including alpha-hemolysin, cytolethal distending toxin, and Shiga-like toxin (Table S5 in the Supplementary material). All of the virulence factors are presumably located on the chromosome except for type 3 fimbriae encoding the gene *mrkABCDF*, which is likely on the plasmid.

## DISCUSSION

Strains 170198^T^ and 170250^T^ were preliminarily assigned to the *Enterobacter cloacae* complex by Vitek II, and 16S rRNA gene sequencing failed to assign exact species. We therefore performed whole-genome sequencing for the two strains. We inferred a core-genome phylogenomic tree comprising type strains of all species belonging to *Enterobacter* and its closely related genera *Huaxiibacter*, *Leclercia*, *Lelliottia*, and *Pseudoenterobacter*. This allowed us to uncover that strains 170198^T^ and 170250^T^ were clustered within the genus *Enterobacter*. Next, we determined the ANI and *is*DDH values. Strain 170250^T^ had the highest ANI (92.93%) and *is*DDH (49.9%) values with the type strain of *E. chengduensis*, confirming that it is of a novel species. Strain 170198^T^ had the highest ANI (95.58%) and *is*DDH (64.4%) value with the type strain of *Enterobacter asburiae*. The 95.58% ANI falls into the 95%–96% inconclusive zone for species demarcation (23, 24), but the *is*DDH values are well below the ≥70% cutoff to define the species (25). Notably, *Enterobacter dykesii* exhibited even a higher ANI (96.37% with the type strain of *Enterobacter muelleri*, with a 69.5% *is*DDH) when it was proposed as a new species (7), and its species status has been validated later (41). Therefore, strain 170198^T^ also represents a novel *Enterobacter* species. The biochemical profile and the whole fatty acid composition of the two that were isolated were generally consistent with those of other *Enterobacter* species with the exception of some specific metabolic characteristics. In addition, the two strains can be differentiated from all known *Enterobacter* species by intricate difference of biochemical characteristics (see the following species description sections).

The two strains caused bloodstream infection, indicating that the two novel species are clinically relevant. We therefore examined the profile of virulence factors of the two strains. The two strains were equipped with a variety of virulence factors for adherence (type I fimbriae and HCP), biofilm formation (curli fibrils, HCP, and type 3 fimbriae), invasion (HCP), and iron acquisition (*iucABCD-iutA* and *ent* genes) (42–46). In particular, aerobactin siderophore encoding the gene *iucABCD-iutA* and enterobactin encoding the *ent* gene cluster, both for iron acquisition, have been found as major virulence factors in *Enterobacter* (44). In addition, the two strains had their respective important virulence factors. Notably, strain 170198^T^ encoded CPS that has been identified as a critical virulence factor for promoting the pathogenesis of *Enterobacter* infections (47). In contrast, strain 170250^T^ had genes encoding hemolysin, cytolethal distending toxin, and Shiga-like toxin, which have been found related to invasion in *Enterobacter* (48). The presence of multiple virulence factors of the two strains corresponds to their pathogenic role in human infection, although the two patients had no septic shock and recovered uneventfully after antimicrobial therapy, indicating the relatively low virulence of the two strains. Nevertheless, compared to the two most common clinically encountered species of *Enterobacteriaceae*, *E. coli* and *Klebsiella pneumoniae*, the virulence of *Enterobacter* is less studied and warrant further exploration.

There are limitations of this study. First, only one strain was obtained for each species. The phenotypic characteristics may vary when more strains are recovered and studied in the future. Second, this study focused on the taxonomy of two strains. Experimental studies of the exact mechanisms for resistance to antimicrobial agents such as fosfomycin resistance in strain 170198 and discrepant susceptibility to several β-lactams between the two strains are beyond the scope of this study but warrants further studies. Likewise, we did not validate the function of predicted virulence genes. Nevertheless, despite the limitations, the characterization of two novel species that are likely opportunistic pathogens for human of clinical relevance.

In conclusion, genotypic and phenotypic characteristics confirmed that strains 170198^T^ and 170250^T^ represent two novel species of the genus *Enterobacter*, for which we propose the name *Enterobacter chinensis* and *Enterobacter rongchengensis*. The type strains is 170198^T^ and 170250^T^, respectively.

### Description of *Enterobacter chinensis* sp. nov.

*Enterobacter chinensis* (chin. en’sis. N.L. masc. adj. *chinensis*, referring to China, where the strain was found).

Cells are Gram negative, 0.8–1.0 μm in size, rod-shaped, motile, and facultatively anaerobic (Fig. S2 in the Supplementary material). Colonies are round, white, translucent, convex, and smooth after incubation overnight at 37°C on nutrient agar. Growth occurs at 10–40°C, and 37°C is the optimum temperature. No growth occurs at 10% (wt/vol) NaCl in TSB. Cells are able to grow in the range of pH 5.0–10.0, and the optimal pH is 6.0–7.0. It has a positive reaction for citrate utilization, β-galactosidase, gelatinase, ornithine decarboxylase, and Voges–Proskauer test, but is negative for arginine dihydrolase, deaminase, H_2_S production, indole production, lysine decarboxylase, and urea hydrolysis. Acid is produced from amygdalin, arbutin, citrate, D-cellobiose, D-galactose, D-glucose, D-lactose (bovine origin), D-maltose, D-mannitol, D-mannose, D-raffinose, D-ribose, D-saccharose (sucrose), D-sorbitol, D-trehalose, D-xylose, D-turanose, esculin ferric citrate, gentiobiose, glycerol, inositol, L-arabinose, L-sorbose, L-xylose, melibiose, methyl-alpha-D-glucopyranoside, N-acetylglucosamine, salicin, and sucrose, but not from adonitol, amidon (starch), amygdalin, D-arabinose, D-arabitol, D-fucose, D-fructose, D-melezitose, D-lyxose, D-tagatose, dulcitol, erythritol, glycogen, inulin, L-arabitol, L-fucose, L-rhamnose, methyl-alpha-D-mannopyranoside, methyl-beta-D-xylopyranoside, potassium gluconate, potassium 2-ketogluconate, potassium 5-ketogluconate, nor xylitol. This species is positive for catalase but negative for oxidase. Colonies do not exhibit hemolysis on blood agar plates. The fatty acid profile is highly similar to type strains of other species of the genus *Enterobacter,* and the major fatty acids are C_16:0_, C_17:0_cyclo, and C_18:1_ω7c.

The type strain is 170198^T^ (= GDMCC 1.3549^T^ = JCM 35826^T^) isolated from a human blood sample at West China Hospital, Chengdu, China.

### Description of *Enterobacter rongchengensis* sp. nov.

*Enterobacter rongchengensis* (rong.cheng.en’sis. N.L. masc. adj. *rongchengensis*, pertaining to Rongcheng, another name referring to Chengdu, China, where the strain was recovered)

Cells are Gram negative, 1.0–2.0 μm in size, rod-shaped, motile, and facultatively anaerobic (Fig. S2). This species forms white, circular, translucent, convex, and smooth colonies on nutrient agar at 37°C after incubation for 24 h. Growth was not observed when the temperature was over 40°C or below 10°C with the optimum growth at 37°C. Growth occurs in TSB containing 0%–9% (wt/vol) NaCl but not in TSB with 10% (wt/vol). Cells grow at pH 5.0–10.0, and the optimal pH is 6.0–7.0. It is positive for arginine dihydrolase, β-galactosidase, gelatinase, ornithine decarboxylase, and Voges–Proskauer reaction, but negative for deaminase, indole production, H_2_S production, lysine decarboxylase, and urea hydrolysis. Amygdalin, citrate, D-cellobiose, D-galactose, D-glucose, D-lactose (bovine origin), D-maltose, D-mannitol, D-ribose, D-sorbitol, esculin ferric citrate, glycerol, inositol, L-arabinose, melibiose, methyl-alpha-D-glucopyranoside, salicin, and sucrose can be metabolized as carbon source to produce acid, while D-trehalose is weakly positive for acidification. No acid is produced by adonitol, amidon (starch), arbutin, D-arabinose, D-arabitol, D-fructose, D-fucose, D-galactose, D-lyxose, D-mannose, D-melezitose, D-raffinose, D-tagatose, D-turanose, D-xylose, dulcitol, erythritol, gentiobiose, glycogen, inulin, L-arabitol, L-fucose, L-rhamnose, L-sorbose, L-xylose, methyl-alpha-D-mannopyranoside, methyl-beta-D-xylopyranoside, N-acetylglucosamine, potassium gluconate, potassium 2-ketogluconate, potassium 5-ketogluconate, nor xylitol. The species is catalase positive and oxidase negative. Colonies do not exhibit hemolysis on blood agar plates. The major whole-cell fatty acids are C_16:0_, C_17:0_cyclo, C_18:1_ω7c, and C_16:1_ ω7c/_C16:1_ω6c.

The type strain is 170250^T^ (= GDMCC 1.3670^T^ = JCM 36189^T^), recovered from a human blood sample at West China Hospital, Chengdu, China.

## Data Availability

The DDBJ/ENA/GenBank accession numbers for the partial 16S rRNA gene sequences of strains 170198^T^ and 170250^T^ are OQ553922 and OQ553924, respectively. The draft whole-genome sequences of strains 170198^T^ and 170250^T^ have been deposited into DDBJ/EMBL/GenBank under accession numbers JARDVI000000000 and JARDVK000000000, respectively.

## SUPPLEMENTAL MATERIAL

Supplemental tables and figures spectrum.00292-24-s0001.docx Tables S1-S5; Fig. S1 and S2.

ASM does not own the copyrights to Supplemental Material that may be linked to, or accessed through, an article. The authors have granted ASM a non-exclusive, world-wide license to publish the Supplemental Material files. Please contact the corresponding author directly for reuse.
