# Supplementary material for: Two novel Enterobacter species, Enterobacter chinensis sp. nov. and Enterobacter rongchengensis sp. nov., recovered from clinical samples carrying multiple virulence factors
Source: Microbiol Spectr. 2024 Jun 25;12(8):e00292-24. doi: 10.1128/spectrum.00292-24 (PMC11302248; doi:10.1128/spectrum.00292-24)
Supplement: Supplemental tables and figures — Tables S1-S5; Fig. S1 and S2. [file spectrum.00292-24-s0001.docx]

Table S1. Biochemical characteristics of the two strains and other *Enterobacter* species

| **Characteristic** | **1** | **2** | **3** | **4** | **5** | **6** | **7** | **8** | **9** | **10** | **11** | **12** | **13** | **14** | **15** | **16** | **17** | **18** | **19** | **20** | **21** | **22** | **23** | **24** | **25** | **26** |
| --- | --- | --- | --- | --- | --- | --- | --- | --- | --- | --- | --- | --- | --- | --- | --- | --- | --- | --- | --- | --- | --- | --- | --- | --- | --- | --- |
| β-Galactosidase | + | + | + | + | + | + | + | + | + | + | + | + | + | + | + | + | + | + | + | + | + | + | + | + | + | + |
| Arginine dihydrolase | - | + | + | + | + | + | + | + | + | + | + | + | + | - | + | + | + | + | + | + | + | + | + | + | + | - |
| Lysine decarboxylase | - | - | + | - | - | - | - | - | - | - | - | - | - | - | - | - | - | + | - | - | - | - | - | - | - | + |
| Ornithine decarboxylase | + | + | + | + | - | + | + | + | + | + | + | + | + | + | + | - | + | + | + | + | + | + | + | + | + | + |
| Citrate utilization | + | + | + | + | + | + | + | + | + | + | + | + | + | + | + | (+) | + | + | + | + | + | + | + | + | + | - |
| H_2_S production | - | - | - | - | - | - | - | - | - | - | - | - | - | - | - | - | - | - | - | - | - | - | - | - | - | - |
| Urea hydrolysis | - | - | + | - | - | - | - | - | - | - | - | - | - | - | - | - | - | + | - | - | - | - | - | - | - | - |
| Deaminase | - | - | - | - | - | - | - | + | - | - | - | - | - | - | - | - | - | - | - | - | - | - | - | nd | nd | - |
| Indole production | - | - | - | - | - | - | - | - | - | - | - | - | - | - | - | - | - | + | - | - | - | - | - | - | - | + |
| Voges-Proskauer reaction | + | + | + | + | + | + | - | W | + | + | + | + | + | - | - | + | + | - | + | + | + | + | + | nd | nd | - |
| Gelatinase | + | + | + | + | - | - | - | - | - | - | - | - | - | - | - | - | - | - | - | - | - | - | - | nd | nd | - |
| D-glucose | + | + | + | + | + | + | + | + | + | + | + | + | + | + | + | + | + | + | + | + | + | + | + | + | + | + |
| D-mannitol | + | + | + | + | + | - | + | + | + | + | + | + | + | + | + | + | + | + | + | + | + | + | + | + | + | + |
| Inositol | + | + | + | + | - | + | W | - | - | + | - | + | - | - | - | - | + | - | - | - | + | + | - | + | + | - |
| D-sorbitol | + | + | + | + | - | + | + | + | + | + | + | + | - | + | - | + | + | + | - | - | + | + | + | + | + | + |
| L-rhamnose | - | + | + | - | + | - | + | + | + | + | + | + | + | - | + | + | + | + | + | + | + | + | + | - | + | + |
| Sucrose | + | + | - | + | + | + | + | + | + | + | + | + | - | + | + | + | + | - | + | + | + | + | + | + | + | - |
| Melibiose | + | + | - | - | - | + | + | + | + | + | + | + | - | - | - | + | + | - | + | + | + | + | + | + | + | + |
| Amygdalin | + | + | + | + | + | + | + | + | + | + | + | + | + | + | + | + | + | + | + | + | + | + | nd | nd | nd | - |
| Arabinose | + | + | + | + | + | + | + | + | + | + | + | + | + | + | + | + | + | + | + | + | + | + | + | + | + | + |
| Potassium gluconate | - | - | nd | - | + | - | + | nd | + | - | - | + | - | + | + | - | nd | + | + | - | - | - | + | nd | nd | nd |
| Methyl-α-D-mannopyranoside | - | - | nd | - | - | - | - | nd | + | + | - | + | - | + | W | + | nd | - | - | - | - | - | nd | nd | nd | nd |
| L-fucose | - | - | nd | - | - | - | - | nd | - | V | - | V | + | - | + | - | + | + | - | - | - | - | - | nd | nd | nd |
| D-arabitol | - | - | nd | - | - | - | - | - | (-) | + | - | - | - | - | (-) | - | - | - | - | - | - | - | - | - | - | nd |
| Dulcitol | - | - | nd | - | - | - | - | nd | W | - | + | - | - | - | + | - | + | - | - | - | - | + | - | nd | nd | nd |
| D-turanose | + | - | nd | - | - | - | + | nd | - | + | - | - | - | W | + | - | W | nd | nd | - | - | + | - | nd | nd | nd |
| Motility | + | + | nd | + | + | - | + | + | + | + | + | + | + | - | + | + | + | + | + | + | + | + | + | + | + | nd |

Species or strains: 1, *E. chinensis* 170198^T^; 2, *E. rongchengensis* 170250^T^; 3, *Enterobacter nematophilus* E-TC7^T^; 4, *E. pseudoroggenkampii* 155092^T^; 5, *E. chuandaensis* 090028^T^; 6, *E. sichuanensis* WCHECL1597^T^; 7, *E. chengduensis* WCHECL-C4^T^; 8, *E. soli* BAA-2102^T^; 9, *E. cloacae* ATCC 13047^T^; 10, *E. mori* LMG 25706^T^; 11, *E.* *bugandensis* EB-247^T^; 12, *E. ludwigii* EN-119^T^; 13, *E. cancerogenus* LMG 2693^T^; 14, *E. asburiae* JCM 6051^T^; 15, *E. hormaechei* NBRC 105718^T^; 16*,* *E.* *xiangfangensis* 10-17^T^; 17, *E. kobei* JCM 8080^T^; 18, *E. oligotrophica* CCA6 ^T^; 19, *E. quasihormaechei* WCHEs120003^T^; 20, *E. wuhouensis* WCHEs120002^T^; 21, *E. quasiroggenkampii* WCHECL1060^T^; 22, *E. quasimori* 090044^T^; 23, *E.* *dissolvens* ATCC 23373^T^; 24, *E. vonholyi* E13^T^; 25, *E. dykesii* E1^T^; 26, *Escherichia coli* ATCC 25922.

+, 90-100% positive reaction; (+), 80-90% positive; -, 0-10% positive reaction; (-), 10–20% positive; W, weakly positive; V, varied; nd, not determined.

Data for type strains of each species excepting *E. chinensis* 170198^T^, and *E. rongchengensis* 170250^T^ are from references [1-15].

Table S2. The detailed biochemical characteristics of 170198^T^ and 170250^T^ according to API 20E and API 50CH kits.

| **Characteristic** | **Strains** | |
| --- | --- | --- |
|  | **170198^T^** | **170250^T^** |
| Indole production | - | - |
| H_2_S production | - | - |
| Urea hydrolysis | - | - |
| Voges–Proskauer reaction | + | + |
| Enzyme activity: |  |  |
| β-Galactosidase | + | + |
| Arginine dihydrolase | - | + |
| Lysine decarboxylase | - | - |
| Ornithine decarboxylase | + | + |
| Deaminase | - | - |
| Gelatinase | + | + |
| Acid production from: |  |  |
| Citrate | + | + |
| D-glucose | + | + |
| D-mannitol | + | + |
| Inositol | + | + |
| D-sorbitol | + | + |
| L-rhamnose | - | - |
| Sucrose | + | + |
| Melibiose | + | - |
| Amygdalin | + | + |
| Glycerol | + | + |
| Erythritol | - | - |
| D-arabinose. | - | - |
| L-arabinose | + | + |
| D-ribose | + | + |
| D-xylose | + | - |
| L-xylose | + | - |
| Adonitol | - | - |
| Methyl-beta-D-xylopyranoside | - | - |
| D-galactose  D-fructose | +  - | +  - |
| D-mannose | + | - |
| L-sorbose | + | - |
| Dulcitol | - | - |
| Methyl-alpha-D-mannopyranoside | - | - |
| Methyl-alpha-D-glucopyranoside | + | + |
| N-acetylglucosamine | + | - |
| Esculin ferric citrate | + | + |
| Salicin | + | + |
| D-cellobiose | + | + |
| D-maltose | + | + |
| D-lactose (bovine origin) | + | + |
| D-saccharose (sucrose) | + | W |
| D-trehalose | + | W |
| Inulin | - | - |
| D-melezitose | - | - |
| D-raffinose | + | - |
| Amidon (starch) | - | - |
| Glycogen | - | - |
| Xylitol | - | - |
| Gentiobiose | + | - |
| D-turanose | + | - |
| D-lyxose | - | - |
| D-tagatose | - | - |
| D-fucose | - | - |
| L-fucose | - | - |
| D-arabitol | - | - |
| L-arabitol | - | - |
| Potassium gluconate | - | - |
| Potassium 2-ketogluconate | - | - |
| Potassium 5-ketogluconate | - | - |

+, positive reaction; -, negative reaction; W, weakly positive.

Table S3**.** Fatty acid profiles of 170198^T^, 170250^T^, and species of the genera *Enterobacter*, *Huaxiibacter*, *Leclercia*, *Lelliottia*, and *Pseudoenterobacter*.

| Fatty acid | 1 | 2 | 3 | 4 | 5 | 6 | 7 | 8 | 9 | 10 | 11 | 12 | 13 | 14 | 15 | 16 | 17 | 18 | 19 | 20 | 21 | 22 | 23 | 24 | 25 | 26 | 27 | 28 | 29 | 30 |  |
| --- | --- | --- | --- | --- | --- | --- | --- | --- | --- | --- | --- | --- | --- | --- | --- | --- | --- | --- | --- | --- | --- | --- | --- | --- | --- | --- | --- | --- | --- | --- | --- |
| C_12:0_ | 2.5 | 1.9 | - | 0.9 | 3.9 | 2.8 | 1.0 | 3.9 | 2.5 | 4.1 | 3.2 | 4.0 | 3.9 | 3.0 | 2.5 | 2.3 | - | 3.7 | 3.8 | 3.5 | 2.0 | 4.3 | 4.5 | 3.1 | 3.0 | 3.7 | 3.6 | 4.5 | 4.9 | 3.6 | |
| C_13:0_ | 0.5 | 0.5 | - | 1.0 | 0.4 | 0.3 | 0.7 | 1.1 | 0.6 | 0.5 | 0.1 | 0.9 | 0.6 | 0.7 | 1.2 | 0.4 | - | 1.4 | 0.3 | 0.4 | 0.8 | 0.4 | 0.7 | 0.5 | 1.1 | 1.3 | 1.3 | 1.0 | 1.2 | 0.4 | |
| C_14:0_ | 7.7 | 9.8 | - | 8.0 | 7.7 | 12.4 | 8.3 | 7.6 | 6.7 | 7.3 | 7.9 | 6.2 | 6.5 | 6.5 | 8.4 | 9.7 | - | 5.6 | 5.8 | 6.3 | 10.9 | 6.4 | 8.8 | 5.5 | 7.6 | 6.0 | 6.7 | 7.2 | 6.4 | 5.6 | |
| C_15:0_ | - | - | - | - | - | - | - | - | - | - | 1.1 | - | - | - | - | - | - | - | 2.0 | 2.3 | - | 2.4 | - | - | - | - | - | - | - | - | |
| C_16:0_ | 33.9 | 30.3 | - | 28.3 | 29.4 | 27.1 | 27.4 | 27.3 | 29.3 | 29.6 | 30.8 | 25.7 | 30.3 | 27.9 | 21.8 | 30.2 | - | 22.7 | 27.8 | 25.0 | 25.2 | 30.21 | 23.2 | 28.5 | 21.6 | 23.7 | 27.3 | 27.3 | 24.1 | 34.2 | |
| C_17:0_ | 2.8 | 2.5 | - | 6.0 | 1.7 | 1.2 | 4.1 | 2.4 | 3.4 | 2.5 | 1.0 | 3.2 | 4.1 | 3.4 | 4.9 | 2.0 | - | 4.0 | 1.6 | 1.8 | 2.5 | 2.0 | 4.2 | 2.2 | 3.8 | 2.1 | 3.1 | 2.7 | - | 2.5 | |
| C_17:0_ cyclo | 15.7 | 22.4 | - | 22.0 | 11.9 | 19.4 | 17.9 | 12.4 | 20.4 | 22.9 | 9.6 | 25.2 | 25.6 | 26.0 | 21.1 | 21.7 | - | 14 | 16.2 | 17.3 | 22.3 | 15.9 | 6.7 | 8.8 | 6.9 | 4.9 | 16.0 | 20.4 | 8.5 | 20.8 | |
| C_18:0_ | 0.9 | 0.5 | - | 0.7 | 0.7 | 0.2 | 0.3 | - | 0.5 | 0.7 | 0.5 | 0.4 | 0.7 | 0.5 | 0.4 | 0.5 | - | 0.3 | 0.6 | 0.6 | 0.2 | 0.8 | - | 0.3 | 0.2 | - | - | - | - | 1.1 | |
| C_18:1_ω7c | 12.7 | 11.6 | - | 8.18 | 21.1 | 12.2 | 17.6 | 16.1 | 14.3 | 16.3 | 22.0 | 15.7 | 12.9 | 13.4 | 20.6 | 15.9 | - | - | 21.9 | 25.5 | 12.9 | 17.0 | 18.3 | 20.4 | 21.6 | - | - | - | 18.5 | 11.5 | |
| C_19:0_ cyclo ω8c | 0.6 | 4.9 | - | 3.5 | 1.5 | 1.4 | 0.8 | - | 6.1 | 3.6 | 0.6 | 6.0 | 5.1 | 7.0 | 4.4 | 5.8 | - | 3.3 | 0.7 | 2.0 | 2.0 | 0.7 | - | 0.1 | 0.1 | - | - | - | - | 0.5 | |
| Sum of  iso-C_15:1_ H/C_13:0_ 3-OH | - | - | - | 2.1 | - | 0.4 | 1.0 | 1.7 | 0.6 | 0.4 | - | 0.7 | 0.6 | 0.9 | 1.5 | 0.3 | - | 1.3 | 0.1 | 0.1 | 1.1 | - | 3.0 | 0.5 | 1.1 | - | - | - | 1.1 | - | |
| Sum of  iso-C_16:1_ I/C_14:0_ 3-OH | 7.7 | 7.8 | - | 8.9 | 9.3 | 12.3 | 7.9 | 8.9 | 6.9 | 6.9 | 7.7 | 6.8 | 6.5 | 6.1 | 6.2 | 6.8 | - | 8.1 | - | - | 8.2 | 9.3 | 13.0 | - | - | - | - | - | 11.0 | 7.7 | |
| Sum of  C_16:1_ω7c/C_16:1_ω6c | 13.5 | 5.9 | - | 5.4 | 9.9 | 9.0 | 9.9 | 16.3 | 6.5 | 5.0 | 14.6 | 4.9 | 2.9 | 3.5 | 5.7 | 4.5 | - | 12.6 | 11.1 | 6.8 | 9.0 | 9.6 | 23.0 | 20.3 | 23.2 | 31.8 | 15.0 | 12.0 | - | 10.6 | |

Species and type strains: 1, *Enterobacter chinensis* 170198^T^; 2, *Enterobacter rongchengensis* 170250^T^; 3, *Enterobacter nematophilus* E-TC7^T^; 4, *Enterobacter pseudoroggenkampii* 155092^T^; 5, *E. chuandaensis* 090028^T^; 6, *E. sichuanensis* WCHECL1597^T^; 7, *E. chengduensis* WCHECL-C4^T^; 8, *E. soli* ATCC BAA-2102^T^; 9, *E. cloacae* ATCC 13047^T^; 10, *E. mori* LMG 25706^T^; 11, *E. bugandensis* EB-247^T^; 12, *E. ludwigii* EN-119^T^; 13, *E. cancerogenus* LMG 2693^T^; 14, *E. asburiae* JCM 6051^T^; 15, *E. hormaechei* NBRC 105718^T^; 16, *E. xiangfangensis* 10-17^T^; 17,*E. kobei* DSM 13645^T^; 18, *E. oligotrophica* CCA6 ^T^; 19, *E. quasihormaechei* WCHEs120003^T^; 20, *E. wuhouensis* WCHEs120002^T^; 21, *E. quasiroggenkampii* WCHECL1060^T^; 22, *E. qausimori* 090044^T^; 23, *E. dissolvens* ATCC 23373^T^; 24, *E. vonholyi* E13^T^; 25, *E. dykesii* E1^T^; 26, *Lelliottia amnigena* LMG 2784^T^; 27, *Lelliottia nimipressuralis LMG* 10245^T^; 28, *Lelliottia jeotgali* PFL01^T^; 29, *Leclercia adecarboxylata* LMG 2803^T^; 30, *Huaxiibacter chinensis* 155047^T^.

-, Not detected or not reported.

Data for type strains of each species excepting the two strains in this study are from references [3, 6, 7, 9, 10, 14-23].

Table S4. Antimicrobial susceptibility of strain 170198^T^ and 170250^T^.

| **Antimicrobial** | **170198** |  | **170250** |  |
| --- | --- | --- | --- | --- |
|  | **MIC**  **(mg/L)** | **Category^$^** | **MIC (mg/L)** | **Category^$^** |
| Amikacin | ≤2 | S | ≤2 | S |
| Ampicillin | 16 | I | ≥128/64 | R |
| Ampicillin-sulbactam | ≤8/4 | S | 128/64 | R |
| Amoxicillin-clavulanate | 32 | R | >64/32 | R |
| Aztreonam | ≤1 | S | ≤1 | S |
| Cefazolin | ≥64 | R | ≥64 | R |
| Cefepime | ≤1 | S | ≤1 | S |
| Cefotaxime | ≤1 | S | ≥8 | R |
| Ceftazidime | ≤1 | S | 8 | I |
| Ceftriaxone | ≤1 | S | 8 | S |
| Cefuroxime | 8 | S | ≥64 | R |
| Cephalothin | ≥64 | R | ≥64 | R |
| Ciprofloxacin | ≤0.25 | S | ≤0.25 | S |
| Colistin | 4 | R | 2 | I |
| Doripenem | ≤0.12 | S | ≤0.12 | S |
| Ertapenem | ≤0.5 | S | ≤0.5 | S |
| Fosfomycin | 256 | R | ≤16 | S |
| Gentamicin | ≤1 | S | ≤1 | S |
| Imipenem | ≤1 | S | ≤1 | S |
| levofloxacin | ≤0.5 | S | ≤0.5 | S |
| Meropenem | ≤0.5 | S | ≤0.5 | S |
| Moxifloxacin^#^ | ≤0.5 | S | ≤0.5 | S |
| Piperacillin | ≤2 | S | 8 | S |
| Piperacillin-tazobactam | ≤2/4 | S | 8/4 | S |
| Tetracycline | 4 | S | 2 | S |
| Ticarcillin^#^ | ≤8 | S | ≥128 | R |
| Tigecycline^#^ | 2 | S | 1 | S |
| Tobramycin | ≤1 | S | ≤1 | S |
| Trimethoprim/sulfamethoxazole | ≥8/152 | R | ≥8/152 | R |

^#^The susceptibility categories were interpreted using the breakpoints of EUCAST.

^$^S, susceptible; I, intermediate; R, resistant

Table S5. Virulence factors and related genes predicted by VFanalyzer from the virulence factor database.

| **VFclass** | **Virulence factors** | **Related genes** | **170198**  **(Prediction)** | **170250**  **(Prediction)** |
| --- | --- | --- | --- | --- |
|  |  |  | **draft (draft)** | **draft (draft)** |
| Adherence | CFA/I fimbriae | *cfaB* | orf02264 | orf00039 |
|  |  | *cfaC* | orf02263 | - |
|  | Curli fibers | *cgsD* | orf00994 | - |
|  |  | *cgsF* | orf00995 | - |
|  |  | *cgsG* | orf00996 | - |
|  |  | *csgA* | orf00992 | orf00123 |
|  |  | *csgB* | - | orf00122 |
|  |  | *csgC* | orf00991 | orf00124 |
|  | E. coli common pilus (ECP) | *ecpA* | - | orf00057 |
|  |  | *ecpC* | orf03870 | orf00055 |
|  | Hemorrhagic E.coli pilus (HCP) | *hcpA* | orf03086 | orf01675 |
|  |  | *hcpB* | orf03087 | orf01676 |
|  |  | *hcpC* | orf03088 | orf01677 |
|  |  | *papC* | orf02101 | orf03433 |
|  | Type I fimbriae | *fimA* | orf01601 | - |
|  |  | *fimC* | orf01599 | orf04589 |
|  |  | *fimD* | orf00210; orf01329; orf03863; orf03890; orf04226 | orf00320; orf01345; orf01533; orf01636 |
|  |  | *fimF* | - | orf04586 |
|  |  | *fimH* | - | orf04587 |
|  |  | *fimI* | - | orf04590 |
|  |  | *fimZ* | - | orf04585 |
|  | Hsp60(Legionella) | *htpB* | orf03428 | - |
|  | LPS O-antigen (P. aeruginosa)(Pseudomonas) |  | orf04575 | - |
|  |  | *orfH* | orf04550 | - |
|  | Polar flagella(Aeromonas) | *flmH* | orf00046 | - |
|  |  | *nueA* | orf01172 | - |
|  | Streptococcal plasmin receptor/GAPDH(Streptococcus) | *plr/gapA* | orf00123; orf00489 | - |
|  | Flagella(Pseudomonas) | *fleR* | orf02541 | - |
|  |  | *fliQ* | orf02537 | - |
|  | Lateral flagella(Aeromonas) | *flgI* | orf02562 | - |
|  |  | *lafC* | orf02572 | - |
|  |  | *lafT* | orf02577 | - |
|  |  | *lfgG* | orf02560 | - |
|  |  | *lfhA* | orf02534 | - |
|  | Type 3 fimbriae(Klebsiella) |  | orf04560 | - |
|  |  |  | orf04562 | - |
|  |  |  | orf04564 | - |
|  |  | *mrkB* | orf04563 | - |
|  |  | *mrkD* | orf04561 | - |
| Invasion | Contact-dependent inhibition CDI system | *cdiA* | orf02038 | - |
|  |  | *cdiB* | orf02037 | - |
|  | EhaB | *ehaB* | orf01752 | orf02780 |
|  | Invasion of brain endothelial cells (Ibes) | *ibeB* | orf00658 | orf01950 |
|  | Flagella(Burkholderia) | *cheB* | orf03577; orf03727 | - |
|  |  | *cheR* | orf03578; orf03726 | - |
|  |  | *cheW* | orf03587; orf03677 | - |
|  |  | *cheY* | orf03576; orf03728 | - |
|  |  | *cheZ* | orf03575 | - |
|  |  | *motA* | orf03590; orf03680 | - |
| Iron uptake | Aerobactin siderophore | *iucA* | orf03740 | orf03030 |
|  |  | *iucB* | orf03741 | orf03029 |
|  |  | *iucC* | orf03742 | orf03028 |
|  |  | *iucD* | orf03743 | orf03027 |
|  |  | *iutA* | orf03744 | orf03026 |
|  | Heme uptake | *chuA* | orf00194 | orf00304 |
|  |  | *chuS* | orf00193 | orf00303 |
|  |  | *chuU* | orf00191 | orf00301 |
|  |  | *ybtX* | orf01494 | - |
|  | Pyoverdine(Pseudomonas) | *pvdH* | orf02892 | - |
|  | Ent siderophore(Klebsiella) |  | orf01496 | - |
|  |  |  | orf01498 | - |
|  |  |  | orf01499 | - |
|  |  |  | orf01500 | - |
|  |  |  | orf01502 | - |
|  |  |  | orf01505 | - |
|  |  |  | orf01506 | - |
|  |  |  | orf01495 | - |
|  |  | *entB* | orf01503 | - |
|  |  | *entF* | orf01501 | - |
|  |  | *fepG* | orf01497 | - |
|  | Enterobactin synthesis(Shigella) | *entC* | orf04539 | - |
|  | Heme biosynthesis(Haemophilus) | *hemC* | orf04047 | - |
|  |  | *hemE* | orf04500 | - |
|  |  | *hemG* | orf01634 | - |
|  |  | *hemH* | orf03027 | - |
|  |  | *hemL* | orf02189; orf04421 | - |
|  |  | *hemN* | orf00190 | - |
|  | Heme transport(Shigella) | *shuV* | orf02114 | - |
|  | Periplasmic binding protein-dependent ABC transport systems(Vibrio) | *viuC* | orf02892 | - |
| Regulation | Alternative sigma factor RpoS(Legionella) | *rpoS* | orf02346 | - |
|  | Carbon storage regulator A(Legionella) | *csrA* | orf02403 | - |
|  | GacS/GacA two-component system(Pseudomonas) | *gacA* | orf03621 | - |
|  | PhoPQ(Salmonella) | *phoP* | orf00089 | - |
|  |  | *phoQ* | orf00088 | - |
|  | RcsAB(Klebsiella) |  | orf02799 | - |
| Secretion system | SCI-I T6SS |  | orf00836 | orf02088 |
|  |  |  | - | orf02087 |
|  |  |  | - | orf02086 |
|  |  |  | orf01561; orf02595 | orf01562; orf02083 |
|  |  |  | - | orf02082 |
|  |  |  | - | orf02081 |
|  |  |  | - | orf02950 |
|  |  |  | - | orf02949 |
|  |  |  | - | orf02948 |
|  |  |  | - | orf02946 |
|  | EPS type II secretion system(Vibrio) | *epsE* | orf01234 | orf03249 |
|  | Flagella (cluster I)(Yersinia) | *flgB* | orf00026; orf03695 | - |
|  |  | *flgC* | orf00027; orf03694 | - |
|  |  | *flgD* | orf00028 | - |
|  |  | *flgE* | orf00029 | - |
|  |  | *flgF* | orf00030; orf03691 | - |
|  |  | *flgG* | orf00031; orf03690 | - |
|  |  | *flgH* | orf00032; orf03689 | - |
|  |  | *flgI* | orf00033; orf03688 | - |
|  |  | *flgJ* | orf00034 | - |
|  |  | *flgK* | orf00035 | - |
|  |  | *flgL* | orf00036 | - |
|  |  | *flgM* | orf00024 | - |
|  |  | *flhA* | orf03572; orf03731 | - |
|  |  | *flhB* | orf03573; orf03730 | - |
|  |  | *flhC* | orf03591; orf03681 | - |
|  |  | *flhD* | orf03592; orf03682 | - |
|  |  | *fliA* | orf03629; orf03683 | - |
|  |  | *fliD* | orf03633 | orf03077 |
|  |  | *fliE* | orf03638 | - |
|  |  | *fliF* | orf03639; orf03724 | - |
|  |  | *fliG* | orf03640; orf03723 | - |
|  |  | *fliH* | orf03641 | - |
|  |  | *fliI* | orf03642; orf03721 | - |
|  |  | *fliJ* | orf03643 | - |
|  |  | *fliL* | orf03645 | - |
|  |  | *fliM* | orf03646; orf03717 | - |
|  |  | *fliN* | orf03647; orf03716 | - |
|  |  | *fliP* | orf03649; orf03714 | - |
|  |  | *fliQ* | orf03650; orf03713 | - |
|  |  | *fliR* | orf03651; orf03712 | - |
|  |  | *fliS* | orf03634 | orf03076 |
|  |  | *fliC* | - | orf03078 |
|  |  | *fliZ* | orf03628 | - |
|  | Hcp secretion island-1 encoded type VI secretion system  (H-T6SS)(Pseudomonas) |  | orf03458 | orf01099 |
|  |  |  | orf03457 | orf01100 |
|  |  | *clpV1* | orf03444 | orf01111 |
|  | T2SS (Yst1)(Yersinia) | *yst1O* | orf01225 | orf03258 |
|  | T2SS(Aeromonas) | *exeD* | orf01235 | orf03248 |
|  |  | *exeF* | - | orf03250 |
|  |  | *exeG* | orf01232 | - |
|  | T4SS effectors(Coxiella) |  | orf01950 | - |
|  |  |  | orf03560 | - |
|  | TTSS (SPI-1 encode)(Salmonella) | *iagB* | orf01800 | orf03260 |
|  | T6SS-I(Klebsiella) |  | - | orf02085 |
|  |  |  | - | orf02084 |
|  |  |  | - | orf02951 |
|  |  |  | - | orf02947 |
|  | T6SS-III(Klebsiella) |  | - | orf02944 |
|  | T6SS-II(Klebsiella) |  | orf02513 | - |
| Toxin | Heat-stable cytotonic enterotoxin | *ast* | orf01976 | - |
|  | Hemolysin HlyA(Aeromonas) | *hlyA* | orf03361 | - |
|  | Hemolysin III(Aeromonas) |  | orf02230 | - |
|  | Phytotoxin phaseolotoxin(Pseudomonas) | *cysC1* | orf02336 | - |
| Acid resistance | Urease(Helicobacter) | *ureB* | orf02082 | - |
|  |  | *ureG* | orf02078 | - |
| Adherence and invasion | EF-Tu(Francisella) |  | orf04028; orf04100 | - |
| Amino acid and purine metabolism | Glutamine synthesis(Mycobacterium) | *glnA1* | orf04425 | - |
| Anaerobic respiration | Nitrate reductase(Mycobacterium) | *narH* | orf00939 | - |
| Antiphagocytosis | Alginate regulation(Pseudomonas) | *algU* | orf02526 | - |
|  | Capsular polysaccharide(Vibrio) | *rmlB* | orf02945 | - |
|  |  | *wbfY* | orf02944 | - |
|  |  | *wbjD/wecB* | orf04551 | - |
|  |  |  | - | orf03817 |
|  |  | *wzb* | orf02885 | orf03818 |
|  | Capsule(Klebsiella) |  | orf01058; orf01059; orf01063; orf01066; orf02884; orf02921; orf02923; orf02930; orf02931; orf02932; orf02936; orf02955 | - |
|  |  | *cpsG_1* | orf02935 | - |
|  |  | *galF* | orf02941 | - |
|  |  | *ugd* | orf02956 | - |
|  |  | *wcaI* | orf02933 | - |
|  |  | *wzb* | orf02922 | - |
|  |  | *wzc* | orf02886 | - |
| Biofilm formation | AdeFGH efflux pump/transport autoinducer(Acinetobacter) | *adeG* | orf00380 | orf00522 |
|  | PNAG (Polysaccharide poly-N-acetylglucosamine)  (Acinetobacter) | *pgaC* | orf02160 | orf03381 |
| Cell surface components | Trehalose-recycling ABC transporter(Mycobacterium) | *sugC* | orf00847; orf04302 | - |
| Efflux pump | AcrAB(Klebsiella) |  | orf01531; orf01646; orf01852 | - |
|  |  |  | orf01647 | - |
|  |  | *acrB* | orf01530; orf02641 | - |
|  | FarAB(Neisseria) | *farB* | orf02410 | - |
| Endotoxin | LOS(Haemophilus) | *gmhA/lpcA* | orf01787 | - |
|  |  | *htrB* | orf00006; orf00768; orf02705 | orf04215 |
|  |  | *kdsA* | orf00959 | - |
|  |  | *kdtA* | orf03926 | - |
|  |  | *lgtF* | orf03925 | orf03557 |
|  |  | *lpxA* | orf03002 | - |
|  |  | *lpxB* | orf03001 | orf03645 |
|  |  | *lpxC* | orf03097 | - |
|  |  | *lpxD* | orf03004 | - |
|  |  | *lpfE* | - | orf03648 |
|  |  | *lpxK* | orf01175 | - |
|  |  | *msbA* | orf01176 | - |
|  |  | *opsX/rfaC* | orf03931 | - |
|  |  | *orfM* | orf02190 | - |
|  |  | *rfaD* | orf03933 | - |
|  |  | *rfaE* | orf02096 | - |
|  |  | *rfaF* | orf03932 | - |
|  |  | *wecA* | orf04553 | - |
| Enzyme | Streptococcal enolase(Streptococcus) | *eno* | orf02322 | - |
| Fimbrial adherence determinants | Fim(Salmonella) | *fimD* | orf01598 | orf04588 |
|  |  | *fimF* | orf01596 | orf04586 |
|  |  | *fimH* | orf01597 | orf04587 |
|  |  | *fimI* | orf01600 | orf04590 |
|  |  | *fimZ* | orf01595 | orf04585 |
|  | Sti(Salmonella) | *stiB* | orf00209; orf01328 | orf01344 |
|  | Stk(Salmonella) | *stkB* | orf03045 | - |
|  |  | *stkC* | orf03046 | - |
| Immune evasion | Capsule(Acinetobacter) |  | orf02950 | orf02996 |
|  | Capsule(Streptococcus) | *rmlA* | orf02947 | - |
|  | Exopolysaccharide(Haemophilus) | *galE* | orf01372 | orf02993 |
|  |  | *galU* | orf00930 | - |
|  |  | *mrsA/glmM* | orf01947 | - |
|  |  | *pgi* | orf04292 | - |
|  | LPS(Brucella) | *acpXL* | orf00047 | - |
| Lipid and fatty acid metabolism | Isocitrate lyase(Mycobacterium) | *icl* | orf04282 | - |
|  | Pantothenate synthesis(Mycobacterium) | *panD* | orf03042 | - |
| Magnesium uptake | Mg2+ transport(Salmonella) | *mgtB* | orf03319 | orf00541 |
| Motility | Flagella(Bordetella) | *motB* | orf03589 | - |
| Nonfimbrial adherence determinants | MisL(Salmonella) | *misL* | orf04428 | - |
| Nutritional virulence | Biotin metabolism(Francisella) | *bioB* | orf01355 | - |
|  | Cysteine acquisition(Francisella) |  | orf04333 | - |
|  | Pyrimidine biosynthesis(Francisella) |  | orf03149 | - |
| Others | MsbB2(Shigella) | *msbB2* | orf03552 | - |
|  | O-antigen(Yersinia) |  | orf02738; orf02934; orf02943; orf04552 | - |
| Protease | Pla(Yersinia) | *pla* | orf00441 | - |
| Quorum sensing | Autoinducer-2(Vibrio) | *luxS* | orf02407 | - |
| Serum resistance | LPS rfb locus(Klebsiella) |  | orf02957 | orf02996 |
|  |  | *rmlD* | orf02946 | - |
| Serum resistance and immune evastion | Capsule(Francisella) |  | orf04142 | - |
|  | LPS(Francisella) |  | orf03790 | - |
| Stress adaptation | Catalase-peroxidase(Mycobacterium) | *katG* | orf04485 | - |
|  | Manganese transport system(Neisseria) | *mntB* | orf02397 | orf00664 |
|  | SodCI(Salmonella) | *sodCI* | orf00268 | - |

Fig S1. Phylogenomic tree inferred by GTDB of strain 170198^T^, 170250^T^, and type strains of the genera *Enterobacter*, *Huaxiibacter*, *Leclercia*, *Lelliottia*, and *Pseudenterobacter*. Bootstrap values (>70%) after 1,000 resamplings are shown by color gradients. Scale, 10% nucleotide sequence divergence.


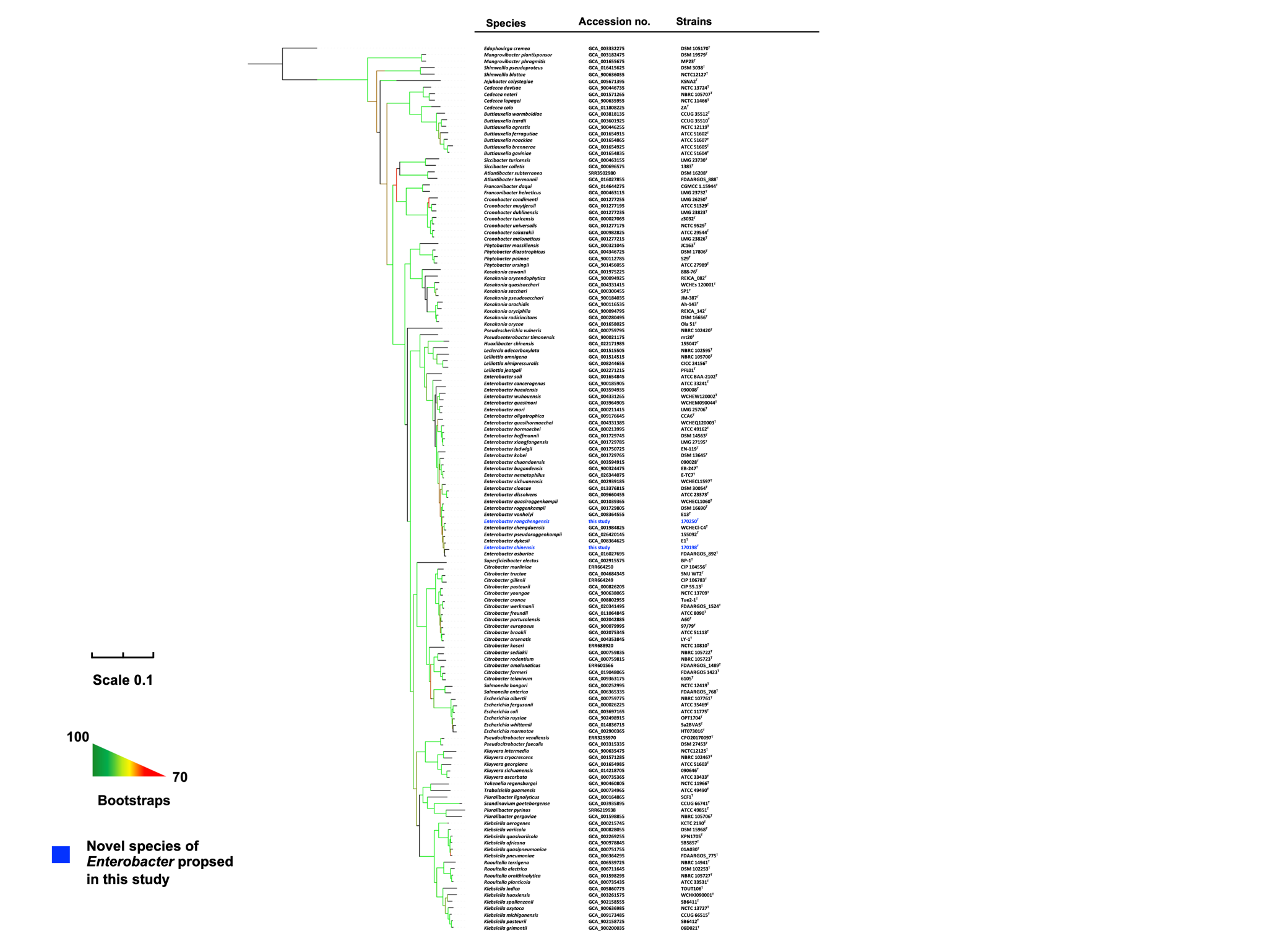


Fig. S2. Transmission electron micrographs of strain 170198^T^ (panel A) and 170250^T^ (panel B) after incubation at 37 °C for 24 h.

**
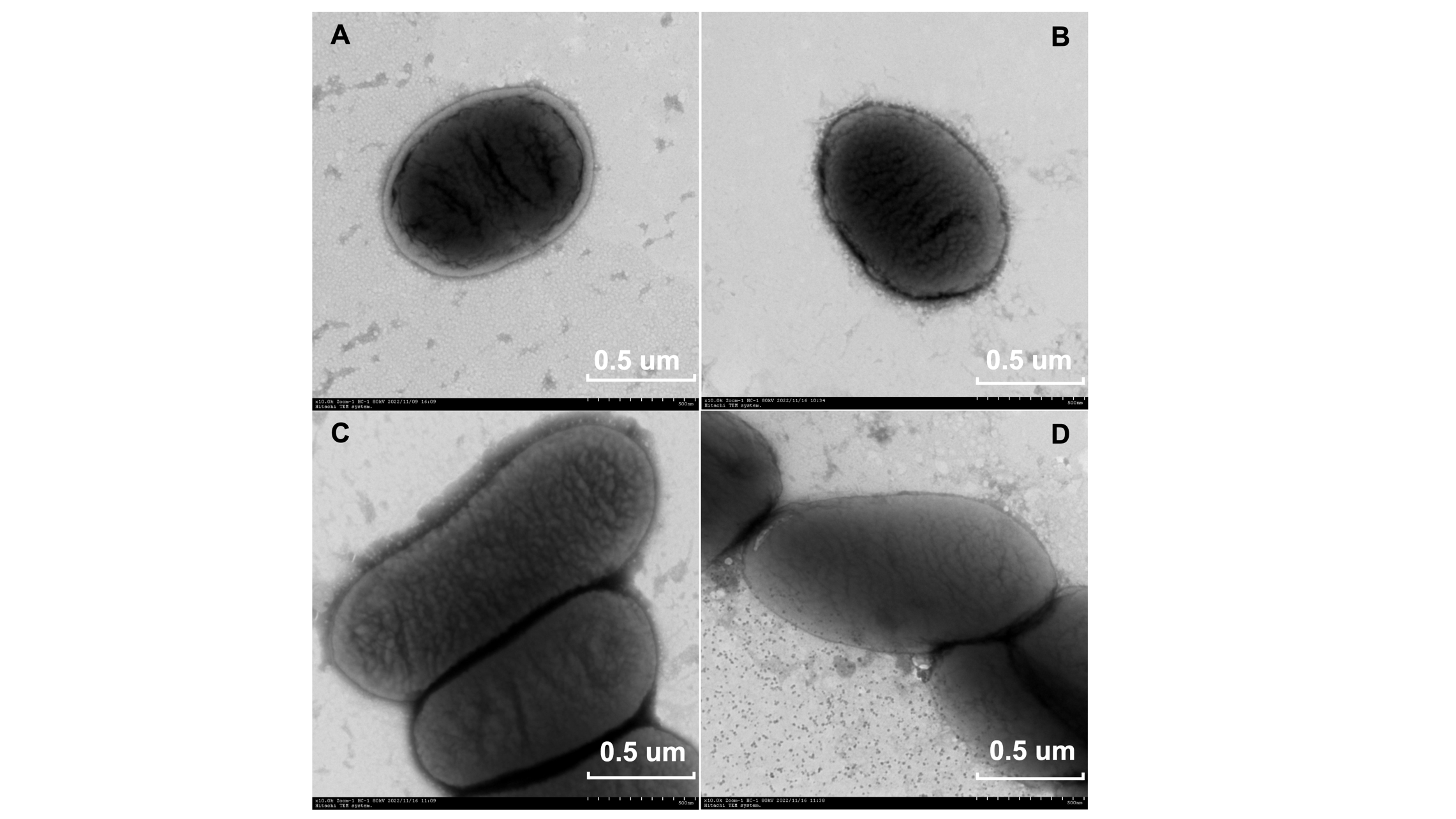
**

**Reference:**

1. Wu, W., et al., *Enterobacter huaxiensis sp. nov. and Enterobacter chuandaensis sp. nov. recovered from human blood.* Int J Syst Evol Microbiol, 2019. **69**: p. 708-714.

2. Akita, H., A. Matsushika, and Z.I. Kimura, *Enterobacter oligotrophica sp. nov., a novel oligotroph isolated from leaf soil.* Microbiologyopen, 2019: p. e843.

3. Wang, C., et al., *Enterobacter wuhouensis sp. nov. and Enterobacter quasihormaechei sp. nov. recovered from human sputum.* Int J Syst Evol Microbiol, 2020. **70**(2): p. 874-881.

4. Wu, W., Y. Feng, and Z. Zong, *Precise species identification for Enterobacter: a genome sequence-based study with reporting of two novel species, Enterobacter quasiroggenkampii sp. nov. and Enterobacter quasimori sp. nov.* mSystems, 2020. **5**(4): p. 00527-20.

5. Hoffmann, H., et al., *Description of Enterobacter ludwigii sp. nov., a novel Enterobacter species of clinical relevance.* Syst Appl Microbiol, 2005. **28**(3): p. 206-12.

6. Cho, G.S., et al., *Polyphasic study of antibiotic-resistant enterobacteria isolated from fresh produce in Germany and description of Enterobacter vonholyi sp. nov. isolated from marjoram and Enterobacter dykesii sp. nov. isolated from mung bean sprout.* Syst Appl Microbiol, 2021. **44**(1): p. 126174.

7. Brady, C., et al., *Taxonomic evaluation of the genus Enterobacter based on multilocus sequence analysis (MLSA): proposal to reclassify E. nimipressuralis and E. amnigenus into Lelliottia gen. nov. as Lelliottia nimipressuralis comb. nov. and Lelliottia amnigena comb. nov., respectively, E. gergoviae and E. pyrinus into Pluralibacter gen. nov. as Pluralibacter gergoviae comb. nov. and Pluralibacter pyrinus comb. nov., respectively, E. cowanii, E. radicincitans, E. oryzae and E. arachidis into Kosakonia gen. nov. as Kosakonia cowanii comb. nov., Kosakonia radicincitans comb. nov., Kosakonia oryzae comb. nov. and Kosakonia arachidis comb. nov., respectively, and E. turicensis, E. helveticus and E. pulveris into Cronobacter as Cronobacter zurichensis nom. nov., Cronobacter helveticus comb. nov. and Cronobacter pulveris comb. nov., respectively, and emended description of the genera Enterobacter and Cronobacter.* Syst Appl Microbiol, 2013. **36**(5): p. 309-19.

8. Izard, D., et al., *Deoxyribonucleic Acid Relatedness Between Enterobacter cloacae and Enterobacter amnigenus sp. nov.* Int J Syst Bacteriol, 1981. **31**(1): p. 35-42.

9. Yuk, K.J., et al., *Lelliottia jeotgali sp. nov., isolated from a traditional Korean fermented clam.* Int J Syst Evol Microbiol, 2018. **68**(5): p. 1725-1731.

10. Brenner, D.J., et al., *Enterobacter asburiae sp. nov., a new species found in clinical specimens, and reassignment of Erwinia dissolvens and Erwinia nimipressuralis to the genus Enterobacter as Enterobacter dissolvens comb. nov. and Enterobacter nimipressuralis comb. nov.* J Clin Microbiol, 1986. **23**(6): p. 1114-20.

11. Steigerwalt, A.G., et al., *DNA relatedness among species of Enterobacter and Serratia.* Can J Microbiol, 1976. **22**(2): p. 121-137.

12. Tamura, K., et al., *Leclercia adecarboxylata Gen. Nov., Comb. Nov., formerly known as Escherichia adecarboxylata.* Curr Microbiol, 1986. **13**(4): p. 179-184.

13. He, Y., et al., *Huaxiibacter chinensis gen. nov., sp. nov., recovered from human sputum.* Int J Syst Evol Microbiol, 2022. **72**(8): p. 005484.

14. Wu, S., et al., *Enterobacter pseudoroggenkampii sp. nov. carrying quinolone-resistant gene qnrE recovered from clinical samples in China.* Antonie Van Leeuwenhoek, 2023. **116**(7): p. 643-651.

15. Machado, R.A.R., et al., *Acinetobacter nematophilus sp. nov., Alcaligenes nematophilus sp. nov., Enterobacter nematophilus sp. nov., and Kaistia nematophila sp. nov., Isolated from Soil-Borne Nematodes and Proposal for the Elevation of Alcaligenes faecalis subsp. faecalis, Alcaligenes faecalis subsp. parafaecalis, and Alcaligenes faecalis subsp. phenolicus to the Species Level.* Taxonomy, 2023. **3**(1): p. 148-168.

16. Wu, W., et al., *Enterobacter huaxiensis sp. nov. and Enterobacter chuandaensis sp. nov., recovered from human blood.* Int J Syst Evol Microbiol, 2019. **69**(3): p. 708-714.

17. Akita, H., A. Matsushika, and Z.I. Kimura, *Enterobacter oligotrophica sp. nov., a novel oligotroph isolated from leaf soil.* Microbiologyopen, 2019. **8**(9): p. e00843.

18. Wu, W., Y. Feng, and Z. Zong, *Precise Species Identification for Enterobacter: a Genome Sequence-Based Study with Reporting of Two Novel Species, Enterobacter quasiroggenkampii sp. nov. and Enterobacter quasimori sp. nov.* mSystems, 2020. **5**(4).

19. Hoffmann, H., et al., *Reassignment of enterobacter dissolvens to Enterobacter cloacae as E. cloacae subspecies dissolvens comb. nov. and emended description of Enterobacter asburiae and Enterobacter kobei.* Syst Appl Microbiol, 2005. **28**(3): p. 196-205.

20. Izard, D., et al., *Deoxyribonucleic Acid Relatedness Between Enterobacter cloacae and Enterobacter amnigenus sp. nov.* International Journal of Systematic and Evolutionary Microbiology, 1981.

21. Steigerwalt, A.G., et al., *DNA relatedness among species of Enterobacter and Serratia.* Can J Microbiol, 1976. **22**(2): p. 121-37.

22. Tamura, K., et al., *Leclercia adecarboxylataGen. Nov., Comb. Nov., formerly known asEscherichia adecarboxylata.* 1986. **13**(4): p. 179-184.

23. He, Y., et al., *Huaxiibacter chinensis gen. nov., sp. nov., recovered from human sputum.* Int J Syst Evol Microbiol, 2022. **72**(8).
